# Targeting General Transcriptional Machinery as a Therapeutic Strategy for Adult T-Cell Leukemia

**DOI:** 10.3390/molecules23051057

**Published:** 2018-05-02

**Authors:** Regina Wan Ju Wong, Takashi Ishida, Takaomi Sanda

**Affiliations:** 1 Cancer Science Institute of Singapore, National University of Singapore, Singapore 117599, Singapore; csirwwj@nus.edu.sg; 2 Division of Hematology and Oncology, Department of Internal Medicine, School of Medicine, Iwate Medical University, Morioka 020-8505, Iwate, Japan; itakashi@iwate-med.ac.jp; 3 Department of Medicine, Yong Loo Lin School of Medicine, National University of Singapore, Singapore 117599, Singapore; 4 14 Medical Drive, Centre for Translational Medicine, #12-01, Singapore 117599, Singapore

**Keywords:** super-enhancer, transcription factor, CDK7, CDK9, BRD4, adult T-cell leukemia

## Abstract

Cancer cells are highly reliant on certain molecular pathways, which support their survival and proliferation. The fundamental concept of molecularly targeted therapy is to target a protein that is specifically deregulated or overexpressed in cancer cells. However, drug resistance and tumor heterogeneity are major obstacles in the development of specific inhibitors. Additionally, many driver oncogenes exert their oncogenic property via abnormal expression without having genetic mutations. Interestingly, recent accumulating evidence has demonstrated that many critical cancer genes are driven by a unique class of enhancers termed super-enhancers. Genes associated with super-enhancers are relatively more susceptible to the inhibition of general transcriptional machinery compared with genes that are regulated by typical enhancers. Cancer cells are more sensitive to treatment with small-molecule inhibitors of CDK7 or BRD4 than non-transformed cells. These findings proposed a novel strategy to identify functionally important genes as well as novel therapeutic modalities in cancer. This approach would be particularly useful for genetically complicated cancers, such as adult T-cell leukemia (ATL), whereby a large mutational burden is present, but the functional consequences of each mutation have not been well-studied. In this review, we discuss recent findings on super-enhancers, underlying mechanisms, and the efficacy of small-molecule transcriptional inhibitors in ATL.

## 1. Introduction

Recent advancements in molecularly targeted therapy have yielded remarkable improvements in outcome for several cancers. Since the success of tyrosine kinase inhibitors such as imatinib and gefitinib in the early 2000s [1,2], a number of small-molecule inhibitors have been developed in the past two decades, many of which have been already tested in clinical trials [3,4]. This approach is supported by the concept that cancer cells are highly reliant on a certain driver oncogene; this phenomenon is termed oncogene addiction or pathway dependence [5,6,7]. Acute inhibition of the pathway that cancer cells depend on for their survival or proliferation results in a dramatic clinical response [2,8]. The application of whole genome and exome sequencing technologies has further provided a molecular understanding of the driver abnormalities in various cancers. However, we have also learned about several major challenges, including drug resistance and tumor heterogeneity. The emergence of resistant clones for biological or pharmacological reasons has occurred for almost all of the drugs that have been developed [9]. The genetic heterogeneity of mutational profiles among patients and the heterogeneity of cancer clones in the same patient or within the same tumor often impede the use of small-molecule inhibitors [10,11,12]. Several cancers associated with particular carcinogens typically exhibit a large number of genetic alterations [13]. Hence, the driver abnormalities could be different among patients. Therefore, in addition to pathohistological examination, further precise and prospective investigation of genetic abnormalities is necessary for the selection of therapeutic drugs [14,15]. Alternatively, it is important to identify and inhibit the molecular pathways that are commonly required for cancer cell survival and proliferation in different cases.

The most fundamental concept of molecularly targeted therapy is to target a protein that is specifically deregulated or overexpressed in cancer cells to avoid non-specific cytotoxicity in non-transformed cells. Thus, many of the small-molecule inhibitors that have been developed to date were designed to directly target an oncoprotein (disease gene). On the other hand, several new drugs that affect general cellular machinery have been recently developed and shown to possess potent anti-tumor cytotoxicity. These new drugs include small-molecule inhibitors of CDK7, CDK9, and BRD4, which block RNA polymerase II or histone activation, leading to transcriptional inhibition [16,17,18]. Although these inhibitors affect general transcriptional machinery, surprisingly, their effects were found to be relatively more specific to cancer cells than to non-transformed cells [16,17,18]. Treatment with these inhibitors results in the acute and concurrent downregulation of multiple oncogenes, thereby resulting in the disruption of various oncogenic mechanisms. Interestingly, recent genome-wide profiling of regulatory elements has shown that the genes that are sensitive to transcriptional inhibition are often regulated under a different class of enhancers called “super-enhancers”, which exhibit a high level of active histone marks [17,19,20]. These findings suggested that the genes required for the maintenance of cancer cells need to be continuously expressed at a high level to sustain their survival and proliferation.

## 2. Deregulation of Enhancers in Cancers

**Figure 1:**
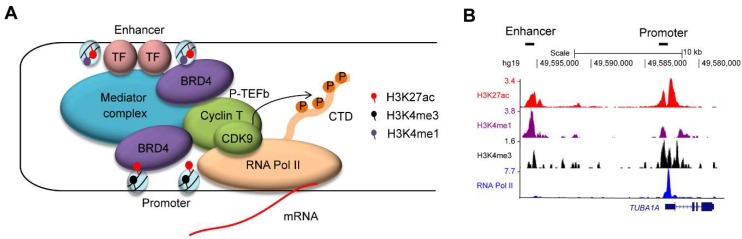
(**A**) Enhancers are bound by transcription factors (TFs). The mediator complex aids in chromatin–chromatin interaction between enhancer and promoter regions. Enhancer and promoter regions are primarily enriched for H3K27ac/H3K4me1 and H3K27ac/H3K4me3, respectively. BRD4 binds to the acetylated lysine residue of histone H3 and recruits the P-TEFb complex (CDK9/Cyclin T), which in turn phosphorylates the C-terminal domain (CTD) of RNA Polymerase II (Pol II) at serine 2 residue. (**B**) An example of chromatin immunoprecipitation-sequencing (ChIP-seq) analysis for histone modifications (H3K27ac, H3K4me1, and H3K4me3) and RNA Pol II in a T-cell acute lymphoblastic leukemia (T-ALL) cell line (Jurkat). The datasets have been reported in Mansour et al. [26], Sanda et al. [36] and Leong et al. [37]. The y-axis represents the total number of mapped reads per million at the *TUBA1A* gene locus. A putative enhancer region enriched for H3K27ac/H3K4me1 and a promoter region enriched for H3K27ac/H3K4me3 are indicated.

Enhancers are regulatory elements that are bound by transcription factors and activate transcription through interactions with promoter elements [21,22,23,24] (Figure 1A). Combinations of the various histone marks that are associated with specific chromatin and transcriptional status have been used to characterize putative enhancer and promoter regions [22,23] (Figure 1B). The application of recent chromatin immunoprecipitation-sequencing (ChIP-seq) technology and chromosome conformation capture assays allowed us to predict regulatory elements on a genome-wide scale in various cell types. Importantly, enhancers are dynamic, changing their activity and landscape during developmental processes or upon environmental changes, and allowing cells to produce a complex pattern of gene expression. As such, the strict and tight regulation of enhancers is necessary for tissue homeostasis. In other words, aberrant functioning of enhancers leads to pathogenesis such as cancer.

The abnormal activation or repression of enhancers is often found in cancer cells, and is closely associated with the misexpression of cancer genes [21,24]. A classic example is the chromosomal translocation involving the *T-cell receptor* (*TCR*) or *immunoglobulin* (*Ig*) gene locus in lymphoid malignancies, which replaces the regulatory element of a translocation partner gene and drives its expression [25]. Many proto-oncogenes, such as *MYC*, *BCL2*, and *TAL1*, were identified from the breakpoints of chromosomal translocation in leukemias and lymphomas [25]. In these cases, the affected proteins are structurally intact, and thus, the overexpression or ectopic expression of wild-type genes can drive carcinogenesis. This is in contrast to many kinase abnormalities, which are often functionally altered due to genetic mutations. In addition to chromosomal translocation, several other mechanisms that activate enhancers have been reported. As a recent example, Mansour et al. identified small nucleotide insertions in non-coding elements upstream of the *TAL1* oncogene in a subset of T-cell acute lymphoblastic leukemia (T-ALL) cases [26]. This insertion creates a binding motif that can be recognized by the MYB transcription factor, leading to the generation of a new and powerful enhancer driving *TAL1* expression. Similarly, mutations or single nucleotide polymorphisms (SNPs) in regulatory elements at the *LMO1* or *LMO2* gene locus have been reported in neuroblastoma and T-ALL cases [27,28,29]. Mutations in the *TERT* gene promoter that enhance its expression have been found in several cancers [30,31,32]. These findings indicate that the aberrant activation of regulatory elements can be a primary driver mechanism for carcinogenesis. Conversely, the regulatory elements of tumor suppressor genes are frequently silenced due to DNA methylation and/or histone modification in various types of cancers [33,34]. Misexpressions or genetic mutations of chromatin modifiers and epigenetic regulators such as *EZH2*, *DNMT3A*, *MLL*, and *ARID1A* are often found in hematological malignancies and solid tumors, leading to global alterations of the gene expression program [34,35]. Therefore, it is crucial to identify the regulatory elements of genes for a molecular understanding of cancer.

## 3. Super-Enhancers in Normal Development and Cancers

**Figure 2:**
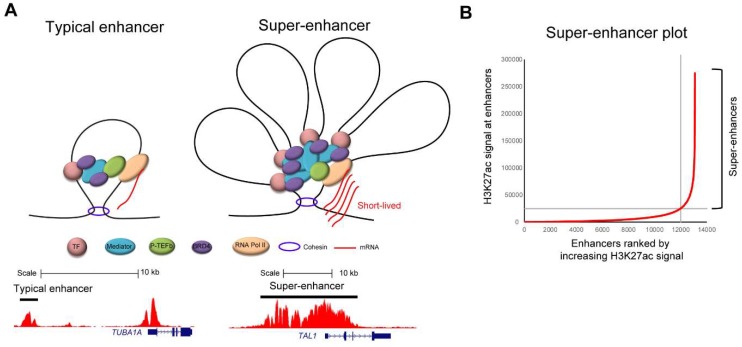
(**A**) Schematic images (top) and examples (bottom) of typical enhancers and super-enhancers. ChIP-seq gene tracks for H3K27ac at *TUBA1A* and *TAL1* gene loci in Jurkat cells are shown. The datasets have been reported in Mansour et al. [26], Sanda et al. [36] and Leong et al. [37]. Black bars represent the putative enhancer elements at each locus. (**B**) A super-enhancer plot showing an example of super-enhancer analysis in an adult T-cell leukemia (ATL) cell line (TL-Om1) analyzed by the ROSE program [17,19,20]. The dataset has been reported in Wong et al. [43]. Briefly, all of the putative enhancer elements are identified by ChIP-seq analysis for H3K27ac. Constituent enhancers are stitched together and then plotted in rank order of increasing H3K27ac signals. Super-enhancers are defined to be those at the right of the inflection point of the curve.

Given the importance of regulatory elements in normal development and pathogenesis, a current area of major research interest is the identification of regulatory elements using genome-wide technologies, such as ChIP-seq [38]. This led to the discovery of a different class of enhancers [17,19,20]. Typically, an enhancer shows a single peak or a few peaks, such as for example by ChIP-seq analysis for H3K27ac (Figure 2A, bottom left). In contrast, there are clusters of enhancers that show significantly high levels of histone marks (Figure 2A, bottom right). Richard Young et al. first described those elements and proposed the new term “super-enhancer” [17,19,20]. Super-enhancers are defined by bioinformatics analysis through ranking all of the putative enhancer elements that are based on ChIP-seq signals, typically for H3K27ac (Figure 2B). However, similar trends can also be observed by ChIP-seq analysis for other enhancer marks (H3K4me1) and mediator proteins or by other methods, including DNase I hypersensitivity assays and Assay for Transposase-Accessible Chromatin using sequencing (ATAC-seq) analysis [20,39]. Although the super-enhancer concept was originally proposed based on bioinformatics analysis, the biological significances of this concept are now being recognized.

In early studies by the Young laboratory, super-enhancers were analyzed in mouse embryonic stem cells (mESCs) and various differentiated cells. They were shown to be associated with critical genes involved in regulating cell fate and identification during normal development [19]. For example, genes encoding pluripotency transcription factors—namely, *Oct4*, *Sox2* and *Nanog*—were associated with super-enhancers in mESCs [19]. In differentiated muscle cells, genes coding the master transcription factor *MyoD* were regulated by super-enhancers [20]. Likewise, the T-cell transcription factor T-bet was driven by super-enhancers specifically activated in T-cells [20]. Similar findings were observed in various types of cells. Interestingly, super-enhancers were also enriched at critical cancer genes, including oncogenes and tumor suppressors. In the catalog of super-enhancers in the 86 cancer cell lines reported by Hnisz et al., super-enhancers were found to be associated with many known oncogenes [19]. In T-ALL cell lines, a large super-enhancer was found at the *TAL1* enhancer locus that was generated by a mutation [18,26] (Figure 2A, bottom right). Super-enhancers were also found at the *GATA3*, *RUNX1*, and *MYB* genes, which are involved in the TAL1-induced core regulatory circuit [36], as well as at the *CDK6* oncogene. The association of super-enhancers with oncogenes is also evident in many other cancers. For example, super-enhancer-associated genes include *IRF4* and *MYC* in multiple myeloma [40], *PAX5*, *MYC*, and *IRF4* in diffuse large B-cell lymphoma [41], *MYCN* and *ALK* in neuroblastoma [42], and *CDK6*, *MYC*, and *EGFR* in glioblastoma [17,20]. These studies suggested that cancer cells may require super-enhancers at key oncogenes to maintain their high level of expression, and allow cancer hallmark traits to be acquired during tumorigenesis. Therefore, the inhibition of super-enhancer activities may serve as a potential therapeutic strategy in various malignancies.

## 4. Targeting General Transcriptional Machinery as a Novel Therapeutic Approach

The strategy of targeting enhancers is ideal for several cancers in which proto-oncogenes are driven by enhancer abnormalities. One approach is to block the binding of responsible transcription factors. However, the “druggability” of transcription factors has been a big challenge since the inception of molecularly targeted therapy. Transcription factors are generally difficult to directly target with small-molecule inhibitors due to insufficient structural information regarding their interaction profiles with one another and their binding modes to DNA [44,45]. Additionally, transcription factors often work together as a complex or work with regulatory partners. As such, the inhibition of a single transcription factor may not be sufficient to modulate transcriptional activity. Therefore, it would be more ideal to target the general transcriptional machinery. However, this suggestion raises a fundamental question. Can the inhibition of general machinery yield a selective block of target genes or specificity of target cells? Recent studies using small-molecule inhibitors of BRD4 and CDK7 have provided several important insights in relation to this question. 

**Figure 3:**
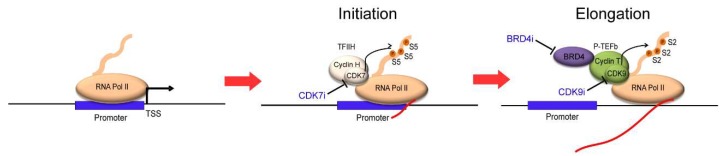
The CTD of RNA Pol II is first phosphorylated by the TFIIH complex (CDK7/Cyclin H) at serine 5 residue to initiate transcription. The P-TEFb complex (CDK9/Cyclin T) further phosphorylates RNA Pol II at serine 2 residue to facilitate transcriptional elongation. Small-molecule inhibitors of BRD4 (BRD4i), CDK7 (CDK7i), and CDK9 (CDK9i) block each of these processes.

The first breakthrough was made by James Bradner et al. in the early 2010s using JQ1, a small-molecule inhibitor of BRD4 [46]. BRD4 belongs to the BET (bromodomain and extra-terminal) family of proteins, and is required to recruit transcriptional elongation factor P-TEFb to acetylated chromatin, which results in the displacement of negative regulators, such as HEXIMI and 7SKsnRNA, from P-TEFb [47] (Figure 1A). The inhibition of BRD4 essentially blocks transcriptional elongation (Figure 3, right). JQ1 has been shown to selectively bind to the amino-terminal twin bromodomains of BRD4 to impair its ability to bind to acetylated histones [46]. Several other BET bromodomain inhibitors, such as I-BET, have also been developed and tested in cancers [48,49]. Importantly, the treatment of these inhibitors causes a remarkable inhibition of the expression of *MYC* and many of the oncogenes that are associated with super-enhancers in various cancers [17,50,51,52]. BRD4 inhibitors preferentially suppress the transcription of cancer-promoting genes compared with housekeeping genes, thereby resulting in the potent cytotoxicity in cancer cells [17,41]. Thus, studies with BRD4 inhibitors indicated that the inhibition of general transcriptional machinery could be a therapeutic option for cancer. 

This idea was further supported by the development of CDK7 inhibitors. CDK7 is a kinase that phosphorylates the C-terminal domain (CTD) of RNA polymerase II at the serine 5 residue, which is required for the initiation of transcription [53] (Figure 3, middle). The inhibition of CDK7 results in the inactivation of RNA polymerase II, leading to a transcriptional block. This effect can be achieved, for example, by using a small-molecule CDK7 inhibitor, THZ1, which was developed by Nathanael Gray et al. [18]. THZ1 covalently binds to the CDK7 protein and irreversibly inhibits its kinase activity, resulting in the reduction of RNA polymerase II CTD phosphorylation. Interestingly, through the screening of over 1000 cell lines, many cancer cell lines were found to be sensitive to THZ1 treatment. As an example, they described that T-ALL cells were very sensitive to CDK7 inhibition; the cell growth of T-ALL cell lines was inhibited at IC_50_ values under 100 nM [18]. T-ALL cells underwent apoptosis in response to low doses of THZ1 treatment, whereas cell cycle arrest was observed in non-transformed BJ fibroblasts and RPE-1 cells at higher doses. This result suggested that normal cells might be less reliant on genes that are sensitive to transcriptional inhibition. Notably, compared with typical enhancer-associated genes, super-enhancer-associated genes, such as *TAL1*, *GATA3*, *RUNX1*, and *MYB* and the oncogene *BCL2*, were more strongly downregulated in T-ALL cells after treatment with THZ1 [18]. In contrast, housekeeping genes, such as *TUBA1A* (encoding α-tubulin), were not associated with super-enhancers (Figure 2A, left), and their expression was hardly affected by THZ1 treatment, even though they were highly expressed. Similar results have been reported in other cancers, including neuroblastoma, small cell lung cancer, and nasopharyngeal carcinoma [42,54,55]. These studies suggested that cancer cells are more sensitive to transcriptional inhibition, and that super-enhancer-associated genes are more preferentially affected after inhibition than typical-enhancer associated genes.

## 5. Possible Mechanisms That Determine Sensitivity to Transcriptional Inhibitors

The aforementioned studies on BRD4 and CDK7 inhibitors highlighted the feasibility of inhibiting the general transcriptional machinery as a therapeutic approach against cancer. However, this result has also raised additional questions. Why are super-enhancer-associated genes more sensitive to these inhibitors than other genes? Is the super-enhancer functionally different from typical enhancers?

Fundamentally, super-enhancers are defined by bioinformatics analysis (Figure 2B). Super-enhancers could be simply the sum of their constituent enhancers, and may not be biologically distinct from typical enhancers. This has been a major debate since this concept was proposed. One plausible explanation is that super-enhancers possess a different chromatin structure and a high density of transcriptional regulators compared with typical enhancers (Figure 2A). In this regard, a phase separation model was proposed to explain the preferential sensitivity of super-enhancers to the inhibition of general transcriptional machinery [56]. Many of the transcriptional components that are present in enhancers can be modified at multiple sites, for example, the phosphorylation or dephosphorylation of the CTD of RNA polymerase II and acetylation or methylation of histones and other transcriptional regulators. Consequently, these chemical modifications allow the components to physically interact with each other, forming “crosslinks”. The formation of such “crosslinks” is therefore dependent on the strength of the chemical interactions between the chemically modified sites. Considering this point, the density of transcriptional components within super-enhancers is remarkably high, possibly allowing the formation of densely connected biomolecular structures that are termed “condensates” through these chemical interactions [57]. Hence, the inhibition of BRD4 or CDK7 would be particularly detrimental to the establishment or maintenance of super-enhancers because of their high “valency”, which is a parameter corresponding to the number of modifiable residues that can affect the engagement of a crosslink. This model implicated the critical roles of BRD4 and CDK7 for the stability of “crosslinks” at super-enhancers.

Another possible explanation is that super-enhancer-associated genes may have different kinetics in transcription. These transcripts could be relatively short-lived, and thus, transcription needs to be continuously activated to maintain their expression. It has been shown that the inhibition of CDK9, which facilitates transcription elongation, primarily affects the accumulation of transcripts with short half-lives, including genes encoding for anti-apoptosis family members and cell cycle regulators [58,59]. Similarly, the inhibition of CDK7 primarily affected mRNA with high turnover rates [60]. Thus, the newly transcribed RNAs (nascent RNAs) are highly dependent on CDK7 and CDK9 and its associated mechanisms, which may require super-enhancers to be continuously transcribed, in order to support cell survival and proliferation. Hence, the inhibition of transcriptional machinery in cancer cells could result in the preferential inhibition of genes that are required for their maintenance.

## 6. Application of Super-Enhancer Profiling and Transcriptional Inhibition to Genetically Complicated Cancer

The development of transcriptional inhibitors provided a new option for cancer therapeutics. Additionally, determination of the susceptibility to transcriptional inhibition can be a novel strategy to identify the critical genes that are required for the maintenance of cancer cells. Early studies on super-enhancers and transcriptional inhibitors were done in T-ALL and neuroblastoma [18,42], which possess a relatively smaller number of genetic and chromosomal abnormalities than other hematological malignancies and solid tumors [13]. One intriguing question is whether the same strategy can be extrapolated to more complicated cancers in which a larger number of abnormalities and greater genetic heterogeneity are prevalent. To test this idea, we recently performed super-enhancer profiling in one of the most intractable cancers, adult T-cell leukemia (ATL), and tested the therapeutic efficacy of CDK7 inhibitors in this disease.

## 7. Molecular Pathogenesis of ATL

ATL is a hematological malignancy derived from T-cells [61,62,63]. There are many marked contrasts between T-ALL and ATL. T-ALL arises from immature thymic T-cell precursors, and is classified under acute leukemia in the World Health Organization (WHO) classification, whereas ATL arises from CD4-positive mature T-lymphocytes, and is classified under mature T and NK neoplasms [64,65]. T-ALL primarily affects young children, whereas ATL mostly occurs in adults of over 50 years old, which is the major limiting factor for the application of high-dose chemotherapy. Additionally, ATL cells are often resistant to conventional chemotherapeutic agents. These characteristics contribute to the dismal prognosis of ATL with the four-year survival rate for the acute-type being less than 20% [66].

Importantly, the transformation process of ATL is highly complicated, and involves multiple mechanisms. The development of ATL is closely associated with the infection of human T-lymphotropic virus type-I (HTLV-1), which is endemic in certain parts of the world, including the southern part of Japan, the Caribbean, and South American and African countries [61]. The viral protein Tax promotes the proliferation of HTLV-I-infected T-cells; however, this gene is often silenced at the diagnosis of ATL, which likely enables the infected cells to escape from host-immune defense [67]. Another important viral gene is *HBZ*, which is continuously expressed throughout the course of the disease and is required for the maintenance of ATL cells and the immunophenotype of HTLV-1-infected cells [68]. Notably, ATL typically arises after 30–50 years of viral infection, and only less than 5% of carriers develop this disease [61,62,63]. Epidemiological studies suggested that the accumulation of multiple genetic and epigenetic abnormalities throughout the latency period would be essential for the complete transformation of infected T-cells [69].

The significance of genetic abnormalities was further elucidated by a recent study using whole genome and exome sequencing technology [70]. Kataoka and Ogawa et al. analyzed mutational profiles in an impressive cohort of 426 ATL samples. Using whole genome sequencing and SNP array-based copy number analysis, they identified approximately 60 structural variations per sample on average, and identified a large number of genetic and chromosomal abnormalities, including activating mutations of *PLCG1* (36%), *PRKCB* (33%), *CARD11* (24%), *VAV1* (18%), *IRF4* (14%), and *CCR7* (11%) in their cohort. They found that many of the mutated genes, such as *PLCG1*, *PRKCB*, *CARD11*, and *STAT3*, are involved in the T-cell receptor (TCR)–NF-κB signaling, T-cell trafficking, and immunosurveillance pathways. The accumulation of additional mutations in genes involved in the TCR-NF-κB pathways, along with the inactivation of *TP53* and *CDKN2A* and other mutations, is likely required for the transformation of T-cells into fully malignant cells.

In addition to genetic abnormalities, several groups have reported the involvement of epigenetic abnormalities in ATL pathogenesis [63]. The *EZH2* gene, which encodes a component of the Polycomb repressive complex 2 (PRC2), was found to be significantly highly expressed and accompanied with enhanced H3K27me3 repressive chromatin marks in ATL cells compared to its normal CD4+ counterparts [71]. Integrative analyses of the epigenome of primary ATL cells by Fujikawa and Watanabe et al. revealed that a global gain of H3K27me3 facilitated by PRC2-mediated epigenetic reprogramming is prevalent in ATL [72]. 

All of these findings indicate that multiple oncogenic mechanisms are involved in ATL. Given the high mutational prevalence of ATL cells, it is difficult to determine the functional property of each mutated gene. Since ATL also exhibits high heterogeneity among patients, it is more reasonable to identify and target molecular pathways that are commonly deregulated across different ATL cases and are functionally important for pathogenesis. Therefore, it would be feasible to apply super-enhancer profiling to identify critical cancer genes and test transcriptional inhibitors in ATL.

## 8. Super-Enhancer Profiles in ATL

In our latest study by Wong and Ngoc et al. [43], we performed super-enhancer profiling for 10 primary ATL samples (nine acute type and one chronic type), and one ATL cell line (TL-Om1) by ChIP-seq analysis for H3K27ac. From this analysis, we identified hundreds of super-enhancers in each sample. We then selected 376 super-enhancer-associated genes that were commonly present in more than five of 10 primary samples. For example, *IL2RA/CD25*, *CD28*, *TNFRSF8/CD30*, *FYN*, and *NFATC1/2* were identified in our study. Interestingly, many of these genes were enriched in the TCR pathway, substantiating the finding by Kataoka et al., which reported that the TCR pathway was often affected by genetic mutations in ATL cells [70].

We then compared this result with profiles in normal T-cells (thymus, Th1, Th2, and Th17) and T-ALL cells. Importantly, three distinct sets of genes surfaced from this analysis. The first set consisted of genes that were commonly associated with super-enhancers in ATL cells, normal T-cells, and T-ALL cells. This set included *CD2*, which has been recognized as a T-cell marker. The second consisted of genes that were associated with super-enhancers in ATL and normal mature T-cells (Th1, Th2, and Th17) but not in normal thymus or T-ALL cells, including the *CD25/IL2RA* gene. This result supported previous findings that ATL arises from CD4+CD25+ mature T-cells [73,74]. Lastly, we found genes that were associated with super-enhancers specifically in ATL samples, but not in normal T-cells. An example was *TP73*, which is a member of the p53 tumor suppressor family. This gene is normally induced upon intracellular stress, such as DNA damage [75,76,77], and is often activated in certain types of solid tumors that exhibit genomic instability [77]. Thus, formation of the *TP73* super-enhancer may reflect the intracellular status of ATL cells. Taken together, our results supported previous findings in other cell types that super-enhancers are enriched in genes that characterize cell identity and fate.

## 9. Application of the Small-Molecule CDK7 Inhibitor

In the same study, we also tested the efficacy of targeting CDK7 kinase in ATL cells [43]. Several ATL cell lines and primary samples were very sensitive to the pharmacological inhibition of CDK7 using small-molecule inhibitor THZ1, even at IC_50_ values below 50 nM. THZ1 treatment efficiently blocked the phosphorylation of RNA polymerase II in sensitive ATL cell lines with a concomitant induction of apoptosis. This study highlighted the feasibility of CDK7 inhibition as a therapeutic option in ATL. Hence, we also performed the gene expression profiling after CDK7 inhibition to find genes that were susceptible to the transcriptional inhibition in ATL cells. We combined this result with super-enhancer profiling to identify potential cancer genes.

Importantly, we have identified the *CCR4* gene to be regulated under super-enhancers in all of the examined ATL samples, but not in normal T-cells or T-ALL cells. The CCR4 protein has been known to be highly expressed in the majority of ATL cases [78,79,80,81]. The expression of *CCR4* was significantly downregulated upon THZ1 treatment, further indicating the involvement of this gene in the pathogenesis of ATL. Additionally, from this analysis, we identified a previously uncharacterized gene, *TIAM2*. This gene was also associated with super-enhancers in all of the examined ATL cases, but not in normal T-cells. The genetic knockdown of *TIAM2* inhibited cell growth and induced apoptosis in ATL cells, whereas the overexpression of *TIAM2* in T-ALL cells promoted cell growth. This result implicated *TIAM2* as a novel candidate cancer gene in ATL. Thus, our approach has successfully demonstrated the potential application of super-enhancer profiling to identify critical cancer genes in genetically complicated cancers.

## 10. Therapeutic Efficacy of the Small-Molecule CDK9 Inhibitor

The use of small-molecule inhibitors of CDK9 has also been explored in ATL and HTLV-1-infected T-cells. The kinase CDK9 is the catalytic subunit of the P-TEFb complex, which activates RNA polymerase II by phosphorylating the serine 2 residue in CTD to facilitate transcription elongation [53] (Figure 3, right). The optimization of a lead compound, BAY 958, which was based on kinase selectivity, physicochemical and drug metabolism, and pharmacokinetics properties, led to the identification of an orally available small-molecule CDK9 inhibitor, BAY 1143572 (atuveciclib; Bayer AG Pharmaceuticals Division, Berlin, Germany) [82]. Currently, this inhibitor is being tested in phase I clinical trials for advanced solid tumors and acute leukemia [83].

Recently, Narita and Ishida et al. investigated the therapeutic efficacy of this CDK9 inhibitor on ATL [84]. BAY 1143572 effectively inhibited CDK9, leading to a reduction in phosphorylation at serine 2 of RNA polymerase II in ATL-derived cell lines and HTLV-1-transformed T-cell lines, as well as in primary ATL samples. Growth inhibition and apoptosis induction were observed and associated with decreased *MYC* and *MCL1* expressions. The administration of BAY 1143572 in immunocompromized NOD/Shi-scid/IL-2Rγ^null^ (*NOG*) mice xenografted with patient-derived ATL cells greatly reduced the infiltration of ATL cells into organs, such as liver and bone marrow. Decreased human soluble *IL2R* levels in serum were also observed, which indicated a reduction of ATL tumor burden. This study revealed the importance of CDK9-mediated transcription in ATL pathogenesis and implicated CDK9 inhibitors as a novel therapeutic agent for ATL.

## 11. Therapeutic Efficacy of the Small-Molecule BRD4 Inhibitor

The small-molecule inhibitor of BRD4 has also been tested in HTLV-1-infected cells. It is noteworthy that the inhibition of BRD4 can exert an additional anti-cancer mechanism besides the inhibition of general transcriptional machinery in ATL or HTLV-1-infected cells. It has been reported that BRD4 mediates the activation of NF-κB. Acetylation of the p65 subunit of NF-κB at lysine 310 and the subsequent recruitment of BRD4 have been shown to be essential for the activation of NF-κB in response to various stimuli [85]. Upon the binding of BRD4 to acetylated p65, CDK9 is recruited to phosphorylate and activate RNA polymerase II for the transactivation of NF-κB target genes. Importantly, NF-κB is often constitutively activated in ATL cells in HTLV-1-dependent and independent manners [86,87]. The viral oncoprotein Tax can activate the NF-κB pathway through the interactions with TAK1 and NEMO/IKKγ [88,89]. Kataoka and Ogawa et al. reported that many of the genes that frequently mutated in ATL are enriched in the T-cell receptor-NF-κB pathway [70]. Our study with super-enhancer profiling also supported this finding [43]. NF-κB can activate cell survival in ATL cells by regulating genes such as BCL2, BCL-xl, and IAP family proteins, for example [90,91]. Therefore, targeting NF-κB provides an ideal therapeutic strategy.

Interestingly, treatment with the BRD4 inhibitor JQ1 has been shown to inhibit proliferation and induce apoptosis in Tax-expressing rat fibroblasts and Tax-positive HTLV-1 infected cells [92]. JQ1 treatment triggered polyubiquitination and the subsequent proteasome-mediated degradation of constitutively active nuclear NF-κB, resulting in the selective inhibition of the expression of NF-κB target genes. JQ1 treatment also resulted in the decreased expression of *IL2RA* and inhibited the growth of HTLV-1 infected cells. Of note, JQ1 also inhibits the proliferation of some Tax-negative HTLV-1 infected cells [92]. This finding indicated the broad application of BET inhibitors in the early stage of HTLV-1 infection, as well as for the treatment of ATL.

## 12. Conclusions and Future Perspectives

Recent mutational profiling by whole genome and exome sequencing has provided a large catalog of mutations in protein-coding genes. However, drug resistance and tumor heterogeneity are major obstacles in the development of specific inhibitors. Additionally, many driver oncogenes exert their oncogenic property via abnormal expression due to enhancer abnormalities, and do not have mutations within protein-coding elements. Therefore, it is critical to determine the driver abnormality that directly contributes to tumorigenesis. This is particularly evident for genetically complicated cancers, such as ATL, whereby a large mutational burden is present, but the functional importance of each mutation has not been well-studied.

In this review, we discussed the therapeutic efficacy of transcriptional inhibitors as well as potential applications of super-enhancer profiling to identify cancer genes. Recent studies suggested that super-enhancers would be required to maintain the expression of critical cancer genes. This would be a common and fundamental mechanism to support cancer cell survival and proliferation, although the acting oncogenes could be different among various cancer types. Given the biological importance of super-enhancers in cancers, super-enhancers represent feasible therapeutic targets. The blocking of super-enhancer-driven transcription resulted in the acute and concurrent disruption of multiple oncogenic machineries to which cancer cells are addicted, regardless of their mutational profiles. Thus, the use of small-molecule inhibitors targeting transcriptional regulators, such as CDK7, CDK9, and BRD4, is an ideal approach. However, we are not optimistic, as no single inhibitor is likely to achieve a maximal anti-cancer effect. A combination treatment with existing therapies is required. Moreover, drug resistance towards THZ1 has already been reported in some cases of *MYCN*-driven neuroblastoma cells in which prolonged treatment with THZ1 resulted in an upregulation of the drug transporters responsible for extruding a variety of drugs [93]. Resistance to JQ1 due to the hyperphosphorylation of BRD4 has also been reported [94]. Hence, a challenge for transforming the use of these inhibitors from lab bench to clinical use will be to improve the specificity of these drugs and overcome the resistance to these agents.
